# Development and Transfer of Microbial Agrobiotechnologies in Contrasting Agrosystems: Experience of Kazakhstan and China

**DOI:** 10.3390/plants14142208

**Published:** 2025-07-17

**Authors:** Aimeken M. Nygymetova, Assemgul K. Sadvakasova, Dilnaz E. Zaletova, Bekzhan D. Kossalbayev, Meruyert O. Bauenova, Jingjing Wang, Zhiyong Huang, Fariza K. Sarsekeyeva, Dariga K. Kirbayeva, Suleyman I. Allakhverdiev

**Affiliations:** 1 Department of Biotechnology, Faculty of Biology and Biotechnology, Al-Farabi Kazakh National University, Al-Farabi 71, Almaty 050038, Kazakhstan; manelaimeken@gmail.com (A.M.N.); asem182010@gmail.com (A.K.S.);; 2 Tianjin Institute of Industrial Biotechnology, Chinese Academy of Sciences, No. 32, West 7th Road, Tianjin Airport Economic Area, Tianjin 300308, China; 3 Ecology Research Institute, Khoja 3Akhmet Yassawi International Kazakh-Turkish University, Turkistan 161200, Kazakhstan; 4 Faculty of Engineering and Natural Sciences, Bahçeşehir University, Istanbul 34349, Turkey; suleyman.allakhverdiev@gmail.com; 5 Institute of Basic Biological Problems, Russian Academy of Sciences, Institutskaya Street 2, Pushchino 142290, Russia; 6 K.A. Timiryazev Institute of Plant Physiology, Russian Academy of Sciences, Botanicheskaya Street 35, Moscow 127276, Russia

**Keywords:** biofertilizers, microbial consortium, technology transfer, Kazakhstan, China, sustainable agriculture

## Abstract

The development and implementation of microbial consortium-based biofertilizers represent a promising direction in sustainable agriculture, particularly in the context of the ongoing global ecological and agricultural crisis. This article examines the agroecological and economic impacts of applying microbial consortiums and explores the mechanisms of technology transfer using the example of two countries with differing levels of scientific and technological advancement–China and Kazakhstan. The analysis of the Chinese experience reveals that the successful integration of microbial biofertilizers into agricultural practice is made possible by a well-established institutional framework that includes strong governmental support for R&D, a robust scientific infrastructure, and effective coordination with the private sector. In contrast, Kazakhstan, despite its favorable agroecological conditions and growing interest among farmers in environmentally friendly technologies, faces several challenges from limited funding to a fragmented technology transfer system. The comparative study demonstrates that adapting Chinese models requires consideration of local specificities and the strengthening of intergovernmental cooperation. The article concludes by emphasizing the need to establish a multi-level innovation ecosystem encompassing the entire cycle of development and deployment of microbial biofertilizers, as a prerequisite for improving agricultural productivity and ensuring food security in countries at different stages of economic development.

## 1. Introduction

Modern agriculture faces a range of interconnected challenges, including rising costs of chemical fertilizers, soil degradation, and a reduction in arable land area. These issues are further exacerbated by global market fluctuations, increasing production expenses, and disruptions in supply chains, all of which contribute to the rising prices of agrochemicals and place a growing financial burden on the agricultural sector [1,2]. Although chemical fertilizers and pesticides offer short-term effectiveness in boosting yields and controlling pests, their widespread use leads to long-term contamination of soil, water, and air, disrupts nutrient cycles, deteriorates soil health, and reduces the diversity of soil microbial communities [3,4,5]. In the long run, this negatively impacts soil fertility, undermines the resilience of agroecosystems, and poses a threat to food security [6]. In response to these challenges, new legislative initiatives, such as the European Green Deal, aim to reduce the use of agrochemicals as a means of ensuring long-term ecosystem sustainability [7].

Against this backdrop of escalating environmental and economic risks, biologically based approaches are gaining prominence. These approaches rely on soil microorganisms capable of enhancing plant nutrition and protecting crops from abiotic stress and diseases [8]. Most commercially available microbial products are based on single-strain formulations; however, this narrow specialization often results in limited adaptability to diverse soil conditions and poor survivability due to competition with indigenous microbial communities [9]. Consequently, their effectiveness in large-scale, long-term field applications is limited. A promising alternative is the use of microbial consortiums (MCs)—synergistic communities of microorganisms that collectively perform a wide array of metabolic and physiological functions [10,11]. The functional advantages of MCs include the activation of key biological processes such as atmospheric nitrogen fixation, solubilization of phosphate and potassium compounds, phytohormone synthesis, micronutrient chelation, and secretion of organic acids [12,13]. These processes not only enhance nutrient availability but also provide comprehensive protection against environmental stresses such as drought, salinity, and pathogenic pressure—factors that are increasingly relevant under changing climatic conditions [10,14].

In addition to improving plant physiological status and enhancing agroecosystem resilience, MCs play a pivotal role in restoring and maintaining soil health. Through the formation of stable symbiotic associations, they improve soil physicochemical properties, facilitate the formation of soil aggregates, regulate water retention, and increase organic matter content [15]. At the same time, MCs stimulate mineralization processes, mobilize nutrients, and improve their bioavailability, all of which directly contribute to agroecosystem productivity [16,17,18]. Of particular interest is their potential in bioremediation: MCs have shown high efficacy in degrading a broad range of contaminants, including polycyclic aromatic hydrocarbons, heavy metals, and petroleum derivatives [19]. Thus, MCs represent a powerful tool for enhancing biological productivity, rehabilitating degraded land, and restoring ecological balance.

However, the high effectiveness of MCs does not guarantee universal applicability without consideration of regional and socioeconomic factors. Successful implementation of such biotechnologies in agricultural practice requires adaptation to specific agroecological conditions, climate-related risks, and the technological development level of a region. This is particularly relevant in the context of global climate change and increasing threats of land degradation and desertification. In this context, a comparative analysis of countries like China and Kazakhstan, each demonstrating distinct models of MC integration in agriculture, is of particular interest. China, with its strong scientific infrastructure and proactive green technology policies, has made significant progress in the development and large-scale deployment of microbial inoculants, including MCs. This has led to increased crop yields and stress tolerance while reducing dependency on chemical fertilizers. Kazakhstan, in turn, continues to face serious challenges, including soil degradation and salinization, water scarcity, and declining land productivity, especially in the southern and western regions of the country. These challenges necessitate the adoption of adaptive and cost-effective solutions, among which biological approaches could play a central role. Their application opens pathways for soil bioremediation, restoration of soil fertility, and improved efficiency in the use of water and mineral resources.

Therefore, comparing the practices of China and Kazakhstan helps identify both universal and locally specific factors influencing the success of biotechnological interventions. This lays the foundation for developing recommendations for adapting and scaling microbial-based products across diverse agroecological zones, particularly in countries facing similar sustainability challenges in agriculture. The aim of this review is to summarize and systematize current scientific knowledge on the application of MCs in agriculture, with a focus on agroeconomic impacts and the specifics of technology transfer between countries with different levels of economic development. The scientific novelty of this work lies in it presenting the first comprehensive comparative analysis of the use of microbial biofertilizers in China and Kazakhstan, including an evaluation of their contributions to agroecosystem productivity and ecological sustainability. The findings may serve as both a theoretical and practical basis for optimizing biotechnological approaches and for developing effective strategies for international scientific and technological cooperation in sustainable agriculture.

## 2. Functional Capabilities of Microbial Consortia in the Context of Sustainable Agriculture

Plants in a favorable physiological state are colonized by a taxonomically structured microbiota that contributes to their growth, nutrient acquisition, stress resilience, and protection against pathogens [20]. Among these microbial communities, plant growth-promoting rhizobacteria (PGPR), residing in the rhizosphere, play a critical role by enhancing nutrient uptake, producing phytohormones, synthesizing hydrolytic enzymes, and generating antimicrobial compounds—thereby improving soil health and supporting plant development under both optimal and stressful conditions [21]. For instance, *Bacillus licheniformis* GDUE.Biol, *B. subtilis* GDUE.Biol, and *B. halotolerans* GDUE.Biol, isolated from the roots of liquorice (*Glycyrrhiza glabra* L.) have demonstrated notable enzymatic activities (protease, amylase, cellulase), antagonistic effects against pathogens, and growth-promoting potential [22].

**Figure 1:**
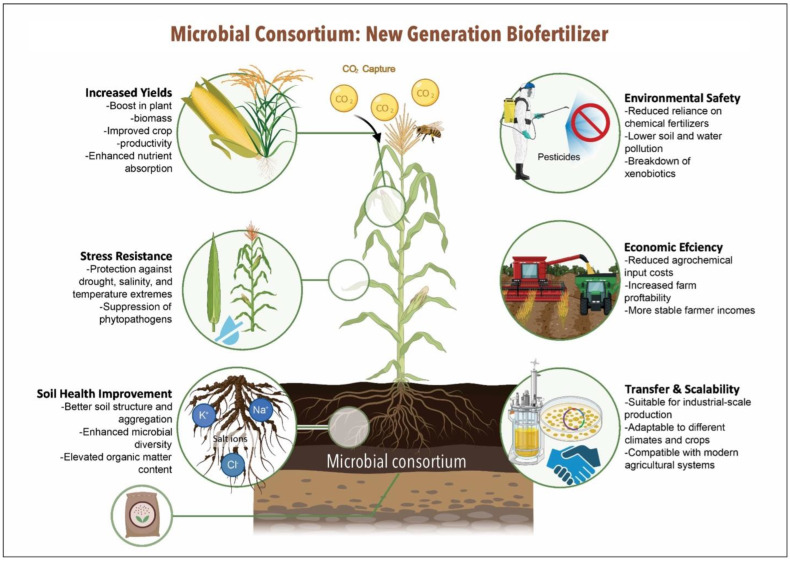
Comprehensive effectiveness of microbial consortia as next-generation biofertilizers.

Given the complexity and microbial diversity of soil ecosystems, there is growing interest in the development and application of MCs as next generation biofertilizers. MCs represent a promising biotechnological platform with the potential to replace conventional agrochemicals and single-strain inoculants, owing to their broad spectrum of complementary metabolic and physiological functions [10,23]. Beyond their multifunctionality, consortia exhibit superior competitiveness against native soil microbiota, enhanced environmental adaptability, and a greater capacity to maintain microbial balance essential for plant health [24]. Figure 1 outlines the key drivers supporting the shift towards MC-based biofertilizers considering current global agricultural challenges. Emerging evidence highlights the versatility and effectiveness of MCs across diverse crop systems and soil conditions, reinforcing their value in sustainable agriculture.

The utilization of MCs exhibiting pronounced antagonistic properties represents a promising strategy for reducing reliance on chemical pesticides while enhancing the environmental safety of agricultural production systems [25]. Consortiums composed of *Trichoderma asperellum* (strains Tricho1, Tricho2) and *Bacillus subtilis* (strains B3, S4KB5, S8KB2) have demonstrated broad-spectrum antagonistic activity against key seed- and soil-borne pathogens, including *Rhizoctonia solani, Fusarium verticillioides, Macrophomina phaseolina, F. udum, F. oxysporum* f. sp. *ciceris, Sclerotium rolfsii, Aspergillus niger,* and *Alternaria sesami,* significantly reducing disease incidence in groundnut and chickpea crops [26].

In addition to their protective roles, MCs offer substantial agronomic and economic benefits by improving plant vigor and increasing crop yields. A consortium comprising *Pseudomonas gessardii* EU-LWNA-25 (phosphorus-solubilizing), *Bacillus* sp. IARI-HHS2-45 (potassium-solubilizing), and *Erwinia rhapontici* EU-B1SP1 (nitrogen-fixing) has shown significant positive effects on amaranth (*Amaranthus hypochondrius* L.) growth, grain formation, and yield compared to both the full NPK fertilizer treatment and uninoculated controls [27]. Likewise, inoculation and co-inoculation with *Bradyrhizobium elkanii* BR 2003, *B. pachyrhizi* BR 3262, *B. yuanmingense* BR 3267, *B. paxllaeri* BR 10398, *B. icense* BR 10399, *Azospirillum baldaniorum* Sp245, and *Bacillus pumilus* UFPEDA 472 significantly enhanced biomass accumulation, nitrogen uptake, and biological nitrogen fixation in mung bean plants, owing to synergistic interactions between the microbial partners and the host plant [28]. Another study confirmed that multifunctional microbial consortiums containing *Bradyrhizobium japonicum* SEMIA 5079 = CPAC 15, *B. diazoefficiens* SEMIA 5080 = CPAC 7, *Azospirillum brasilense* Ab-V5 and Ab-V6, *Bacillus megaterium* CNPMS B1119, *B. subtilis* CNPMS B2084, and *Trichoderma harzianum* Simbi-T5 improved soybean (*Glycine max*) productivity under controlled growth chamber, greenhouse, and field conditions. Although a high microbial load and the presence of *T. harzianum* slightly delayed germination and early vegetative growth, seed yield and lipid content were not adversely affected. These findings underscore the importance of careful strain selection in consortium design to mitigate early-stage negative effects on plant development [29].

In this context, synthetic microbial consortiums (SMCs), rationally assembled based on synergistic interactions and systems biology tools, offer a high-precision strategy for modulating the structure and function of microbial communities. Such consortiums are engineered to perform specific tasks including biocontrol, bioremediation, and nutrient mobilization [30,31,32]. The synthetic community (SynCom) concept is a rapidly emerging field that integrates synthetic biology with metagenomic and bioinformatic insights derived from next-generation sequencing technologies [33]. In the study by Li et al., a synthetic microbial system was developed using genetically engineered *Escherichia coli* strains incorporating two unidirectional modules, enabling the construction of programmable consortiums with tunable ecological interactions, such as synergy, competition, and exploitation, thereby offering a robust experimental platform for exploring microbial community dynamics under controlled conditions [34].

Despite their significant potential, the widespread application of MCs in agriculture remains constrained by technological and logistical limitations. Critical challenges include maintaining microbial viability during storage, transport, and field deployment, as well as tailoring formulations to specific agroecological contexts [35]. These limitations are being addressed through the development of innovative cultivation and formulation strategies. Advances in strain selection, carrier optimization, and preservation techniques have markedly improved shelf life and the stability of biological products [36,37]. Delivery technologies such as encapsulation and seed coating ensure targeted microbial introduction into the rhizosphere, enabling consistent colonization and activity [38]. Optimized cultivation parameters, including nutrient enhancement, adaptive evolution, and bioreactor engineering, boost microbial productivity, stability, and functional metabolite synthesis [39,40]. Formulation improvements, such as the supplementation of specific media components and trace elements (e.g., molybdenum, boron, manganese, magnesium), have been shown to enhance microbial viability and metabolic efficiency [41]. Furthermore, the use of alternative, cost-effective substrates like seaweed waste and microalgal biomass cultivated on wastewater enhances the sustainability of biofertilizer production [36,37]. Carrier matrices such as dolomite and biochar have also proven effective in maintaining microbial viability and prolonging shelf stability [42,43]. The successful scale-up and deployment of microbial biofertilizers necessitate the integration of process engineering, resource efficiency, and microbial ecosystem management to ensure product consistency and efficacy under field conditions [44,45]. Concurrently, the ecological succession and interspecies interactions within MCs must be carefully considered and strategically managed [46].

Ultimately, the transition from experimental applications to commercial-scale deployment of MCs will require a systems-level approach grounded in fundamental research, biotechnological innovation, regional customization, and sustained interdisciplinary collaboration supported by long-term funding frameworks.

## 3. Scientific Foundations and Applied Research on MCs in China

### 3.1. Microbial Biotechnologies in Chinese Agricultural Modernization

The development of agricultural technologies in China began in ancient times, alongside the societal transition from the Stone and Bronze Ages to the Iron Age. This period was marked by important technological advancements, including the invention and gradual adoption of iron farming tools, which significantly improved labor productivity and the quality of soil cultivation. In parallel, various cropping systems were introduced and refined, contributing to the early advancement of agricultural production in China [47]. Over the course of several millennia, Chinese agriculture predominantly relied on organic methods to maintain soil fertility. Green and organic fertilizers, enriched with beneficial microorganisms, played a central role in preserving nutrient balance and preventing soil degradation. These practices served as the foundation of sustainable farming systems across China until the 1960s [48]. A key ideological element in Chinese agrarian history was the principle that “food is the primary need of the people,” first articulated during the Han dynasty (202–220 BCE). This concept emphasized the necessity of stable and efficient agriculture, which was closely linked to the rational use of land and the application of biological approaches to improve soil fertility. Throughout history, agricultural technologies were highly valued within Chinese governance structures, and farmers occupied an esteemed position in the social hierarchy [49]. Historically, China has benefited from a strong foundation for agricultural development, shaped by deep traditions of intensive land use and a systematic approach to biologically supported soil fertility. However, in the contemporary era, the country faces serious challenges, including soil degradation, water scarcity, and the overapplication of agrochemicals [50]. These issues highlight the need for innovative solutions and the continued advancement of biotechnological approaches to ensure sustainable agricultural development [51].

The formation of China’s modern scientific and technological system has been a complex and gradual process, beginning with the reforms of the early 20th century. Although some progress was achieved during the Republic of China era (1912–1949), further advancement was constrained by political instability and limited funding. It was only after the establishment of the People’s Republic of China in 1949 that a systemic restructuring of the scientific sector began, modeled on the Soviet system, which catalyzed the development of a national research infrastructure. A significant surge occurred in the 1990s with the implementation of the “Science and Education as the Foundation of the Nation” strategy, leading to unprecedented state investment in R&D and enhancing China’s standing on the global scientific stage [52].

Within this context, agricultural science emerged as a key area of development, with agroecology evolving into a central focus. As a distinct academic discipline, agroecology has integrated ecological, social, and economic dimensions of agroecosystem functioning. Since the late 1970s, principles of sustainable agriculture have been actively promoted, thanks in part to the foundational works of agroecological theory [53]. In the 1980s, the concept of agroecological engineering was proposed, and the term “ecological agriculture” was introduced into scientific use, thereby laying the foundation for the development of low-energy and sustainable farming methods in China. These ideas were institutionalized in national sustainable development programs, including the creation of “ecological counties” (1993) and the implementation of the “three rural issues” (agriculture, rural areas, and farmers) strategy in the 2000s [54]. In the early 21st century, particularly following the adoption of the 14th Five-Year Plan, the bioeconomy began to be viewed as a strategic driver of the “third biotechnology revolution,” rooted in the integration of molecular biology, microbiology, and genetic engineering into agricultural production systems [55]. Agricultural science thus acquired strategic importance, becoming one of the pillars of technological transformation within China’s agri-industrial complex [56].

The rethinking of traditional agroecosystems through the lens of sustainability has also garnered international recognition. For example, the “rice-fish” system from Qingtian County was the first Chinese agroecological model recognized by the FAO under the GIAHS (Globally Important Agricultural Heritage Systems) program. A national competition for the conservation of agricultural heritage has become a major mechanism for promoting ecologically oriented farming models and preserving biodiversity [57].

One of the most significant directions in China’s 21st-century science and technology policy has been the creation of an integrated agricultural innovation system. A key component of this system has been the establishment of Science and Technology Innovation Centers (STICs) in agriculture. These centers aim to strengthen the interface between science, technology, and the economy, while also serving as platforms for accelerating agricultural R&D and achieving strategic autonomy in the development of high-tech sectors [58]. The conceptual design of STICs involves the analysis of both international and domestic experience, the identification of institutional and technological barriers, and the development of proposals to improve innovation governance, logistics, and spatial coordination [59].

In parallel with the development of STICs, since 2001 China has been implementing a program for establishing national agricultural science and technology parks. Over the past two decades, these parks have evolved into regional agro-innovation hubs. They play a pivotal role in demonstrating new crop varieties, agronomic techniques, and production models, thereby enhancing yields, technological capacity, and farm incomes [60]. The 13th Five-Year Plan provided critical political impetus to these initiatives, emphasizing the need to diversify the agricultural sector, support biotechnological innovations, and modernize traditional production systems through innovation [61].

Although environmentally friendly technologies are being actively promoted, the overuse of chemical pesticides in China remains a significant challenge. Between 1990 and 2016, pesticide use increased nearly 2.5-fold, reaching more than 1.7 million tons [62]. In response, efforts to implement microbial pesticides have intensified, supported by government policies, scientific advancements, and demand for “green” technologies. The development of this sector has a long history: as early as 1959, biological control agents were included in official national reference documents, and by 1975, they were institutionalized under the framework of integrated pest management. Since 2011, microbial pesticides have been systematically incorporated into crop protection protocols, particularly for cereals, and have been prioritized in demonstration projects on green agriculture [63]. As of 2021, over 2900 enterprises in China were involved in this sector. The registry of the Ministry of Agriculture lists 9414 microbial products, with a notable shift from mono-strain to multi-strain formulations to improve the functional stability and adaptability of biopreparations [64].

Chinese state policy continues to actively support environmentally safe technologies, including the development and deployment of MCs, subsidies for innovative solutions, and the establishment of agrotechnology parks. These measures aim to reduce dependence on chemical pesticides, enhance the resilience of agricultural ecosystems, and promote resource-efficient production [65]. A major milestone in this trajectory has been the advancement of synthetic biology, which received strong support in 2014 with the launch of the “973 Project”. The project’s goal was to design and construct SMCs for applications in industrial biotechnology, bioremediation, and agriculture [66]. In 2018, the Ministry of Science and Technology launched a national key R&D program, and by 2022, synthetic biology had been officially incorporated into the country’s bioeconomy strategy. In 2023, it was designated a technology subject to export restrictions, underscoring its importance for national security and technological sovereignty [67].

Thus, China’s biotechnological trajectory reflects a deliberate transition from adaptive agricultural solutions to the creation of advanced, high-tech biosystems that integrate cutting-edge scientific approaches into both production and environmental contexts.

### 3.2. Scientific Research and Achievements in the Application of MCs in Chinese Agricultural Practice

Since 2003, the People’s Republic of China has systematically issued directives promoting the search for biological alternatives to agrochemicals. A major milestone was the “Zero Growth Action Plan for Fertilizer and Pesticide Use by 2020” (2015), which significantly increased national grant funding for research on biofertilizers and microbial pesticides. The scientific community responded with an exponential growth in publications, firmly establishing MCs as a core technological component of China’s green agricultural agenda [68].

Numerous studies by Chinese researchers have shown that certain plant-associated microorganisms can enhance resistance to plant diseases, leading to growing interest in the development and application of MCs as effective plant protection agents (Table 1).

In addition to reducing biotic stress, MCs containing beneficial microbes can also increase plant tolerance to various abiotic stresses, such as drought, salinity, and heavy metal (HM) toxicity. Among these, HM-induced stress is considered one of the most acute environmental and agricultural challenges in China today. According to a national study conducted in 2014, about 19% of China’s agricultural land is contaminated with heavy metals, particularly arsenic (As), cadmium (Cd), chromium (Cr), copper (Cu), nickel (Ni), lead (Pb), zinc (Zn), and mercury (Hg), while 82.8% of soil samples containing heavy metals exceeded the maximum permissible concentrations [76].

The most critical concern related to HM contamination is the entry of toxic concentrations into human food chains. Rice, a staple crop in China, is especially prone to Cd accumulation. Studies show that a significant proportion of domestically grown rice contains Cd levels exceeding the national food safety limit (0.2 mg kg^−1^), particularly in southern regions [77]. These findings have triggered widespread public and scientific concern regarding food safety and human health, highlighting the urgent need for effective and sustainable solutions to reduce HM concentrations in crops. Recent studies have examined how MCs can reduce HM toxicity and enhance plant tolerance to polluted soils. For example, a consortium of *Pseudomonas* sp. 4N2 and *Bacillus* sp. TB1 enhanced Cd immobilization in rice roots, reducing translocation to shoots (from 30% to 6% in low-accumulating and from 31% to 13% in high-accumulating rice varieties), while also boosting antioxidant activity, exopolysaccharide secretion, and rhizosphere microbial diversity [78]. Another study found that co-inoculation with *Enterobacter* and *Comamonas* reduced Cd levels in rice grains by immobilizing the metal in soil, colonizing the rhizosphere and plant tissues, activating defense signaling pathways, and enhancing enzyme activity involved in Cd binding [79]. Inoculation with *Bacillus licheniformis* P8_B2 and *Pseudomonas aeruginosa* NBRC 12689 significantly reduced Cd in soil pore water and As in roots and shoots (by 17–37%), while also reducing oxidative stress and improving plant growth [80].

Salinity stress also poses a major problem for millions of hectares of arable land in China, especially in northern provinces, the Yellow and Yangtze River deltas, and the arid western regions where high evaporation and intensive irrigation accelerate soil salinization. Drought, likewise, causes substantial annual economic losses by reducing yields of key crops such as rice, wheat, and maize. As a result, the investigation of plant tolerance to HM, salinity, and drought is becoming increasingly significant in the context of climate change and land deterioration (Table 2).

In recent years, China has actively pursued studies on MCs as biofertilizers to promote crop growth. For instance, a probiotic consortium of eight *Pseudomonas* strains (*P. fluorescens* 1m1–96, *P. fluorescens* mvp1–4, *P. fluorescens* Phl1c2, *P. fluorescens* Q2–87, *P. kilonensis* F113, *P. protegens* Pf-5, *P. protegens* CHA0 and *P. brassicacearum* Q8r1-96) significantly improved tomato growth and rhizosphere bacterial density compared to less diverse communities [94]. Additionally, *Bacillus velezensis* FH-1 and *Brevundimonas diminuta* NYM3 regulated rhizosphere microbiome structure and complexity, enhancing rice growth through both direct and microbiome-mediated mechanisms [95]. In a field trial on tea plant (*Camellia sinensis)*, the application of the TCM consortium, which included strains that promote nutrient cycling and restructure the rhizobacterial network, resulted in improved plant growth, such as increased bud density, leaf area, bud weight, and significantly enhanced soil properties. These improvements included an increase in organic matter (60.89%), total nitrogen (66.22%), phosphorus, potassium, and water-stable aggregates. Additionally, the microbial community composition was altered in favor of beneficial phyla and genera, such as *Proteobacteria, Nitrospirae, Hirschia,* and *Nocardioides* [96]. Three years of field observations confirmed the stable effectiveness of a biocontrol drug based on a consortium of *Bacillus subtilis* znjdf and *Trichoderma harzianum* znlkhc1, which contributed to increased yields, accompanied by an increase in the number of functional bacteria and increased activity of genes associated with carbon and nitrogen metabolism [97].

These data collectively highlight the high practical value of MCs as innovative biofertilizers aimed at sustainably increasing crop productivity, improving soil microbial community structure, and supporting environmentally safe agriculture. Beyond biofertilization, MCs have emerged as effective tools for rehabilitating degraded agricultural land in China. The country faces critical challenges due to the accumulation of residual agrochemicals, especially herbicides, insecticides, and organophosphates, that degrade soil quality, reduce biological activity, and ultimately threaten food security. In this context, MCs are seen as eco-friendly and efficient solutions for bioremediation and agroecosystem restoration. Their synergistic microbial interactions enable the degradation of xenobiotics and restoration of soil microbiomes, making previously unusable lands arable once again. The YM1 bacterial consortium (*Bacillus cereus* N1, *B. amyloliquefaciens* Mq4, *Serratia marcescens* N80, and *Klebsiella jilinsis* 2N3) achieved up to 95.5% degradation of nicosulfuron in contaminated soil. The mitigation of herbicide-induced stress was primarily mediated by enzymatic processes driven by extracellular enzymes [98]. The enriched microbial consortium YS622, consisting mainly of *Azospirillum, Cloacibacterium*, and *Ochrobactrum*, formed from soils contaminated with glyphosate, demonstrated high efficiency in its biodegradation (up to 100% at 50–60 mg/L in 36 h) in aquatic and soil systems, metabolizing it through the aminomethylphosphonic acid pathway [99]. Another enriched MF0904 consortium, comprising *Pandoraea, Stenotrophomonas,* and *Paracoccus*, achieved complete degradation of the carbamate pesticide methomyl in both sterile and natural soils within 72–96 h [100]. Highly efficient biodegradation of acephate in soil systems has been achieved using the ZQ01 microbial consortium, which is capable of degrading more than 80% of the compound within 32 h, over a wide range of temperatures and pH conditions. The genera *Sinomonas, Pandoraea,* and *Burkholderia-Caballeronia-Paraburkholderia* play a key role in this process [101]. Inoculation with a bacterial consortium enriched from β-cyflutrin-contaminated soil and represented mainly by the genera *Enterobacter, Microbacterium, Ochrobactrum, Pseudomonas, Hyphomicrobiaceae*, and *Achromobacter* contributed to an effective reduction in pollution levels in the aquatic and soil environment, reducing the residual content of the pesticide in alfalfa (*Medicago sativa)* and reducing its phytotoxic effects on the plant [102]. These results illustrate that MCs in China have progressed beyond lab-scale trials into practical ecosystem-level applications. Future integration of such technologies into agricultural systems will simultaneously support ecological safety, improve crop productivity, and restore degraded soils.

The development of MCs in China is part of a broader national strategy for transitioning to sustainable agriculture by reducing dependence on agrochemicals. Policy emphasis has been placed on biological innovations that promote ecological farming and restore soil fertility. Substantial funding has been allocated to applied agricultural biotechnology research through major national initiatives such as the National Key R&D Program of China, where microbial fertilizers and biocontrol technologies are prioritized.

Leading national institutions, including the Chinese Academy of Agricultural Sciences (CAAS), Chinese Academy of Sciences (CAS), Beijing Forestry and Agriculture University, Huazhong Agricultural University, and Nanjing Agricultural University, have established a robust research and development platform for the creation and standardization of MCs. In recent years, they have developed a wide range of multifunctional microbial products tailored to China’s diverse soil and climatic conditions. China’s experience in scientifically grounded and large-scale integration of MCs into agricultural practice has become a model for effective agricultural biologization. Unlike fragmented efforts observed in many countries, China has built a cohesive system where research, policy, and practice operate as mutually reinforcing components. This model not only consistently reduces anthropogenic pressure on agroecosystems but also creates export potential for biotechnology, positioning China as a global leader in the transition toward a bio-oriented agricultural economy. In this context, MCs are no longer merely auxiliary tools—they have become the foundation of a new agricultural paradigm: ecological, economically viable, and scientifically advanced.

## 4. The Development and Application of Microbial Fertilizers in Agricultural Practices in Kazakhstan: A Current State and Scientific Perspectives

Kazakhstan’s agriculture has undergone three major phases of development. The first phase (late 19th–early 20th century) was shaped by agrarian transformation under the colonial policies of the Russian Empire, including the resettlement of Russian peasants into the steppes and the forced sedentarization of nomadic populations. The second phase (1920s–1990s) encompassed Soviet collectivization, the Virgin Lands campaign, and post–World War II recovery. The third phase, spanning the post-Soviet era from the 1990s to the present, has been characterized by a transition to a market economy and modernization of the agricultural sector [103].

Kazakhstan’s agrarian sector possesses substantial potential due to its vast arable land resources, agroclimatic diversity, and a rich history of farming practices. Agriculture continues to play a vital role in the national economy by ensuring food security and providing employment for a significant portion of the population [104]. However, traditional farming methods inherited from the past are proving increasingly inadequate in the face of climate change, soil degradation, and global market competition. These challenges are particularly pronounced in steppe and arid regions, where the implementation of adaptive and resource-efficient agrotechnologies is not only relevant but imperative [105].

In the context of global environmental and economic shifts, the introduction of innovations into agricultural practice has gained critical importance. Sectoral modernization is being guided by key national policy frameworks such as “Agrobusiness-2020,” the Agricultural Development Program for 2017–2021, and the Green Economy Transition Concept. These initiatives aim to establish a regulatory and investment environment conducive to the adoption of biotechnologies and advanced agricultural solutions [106,107]. One of the most promising directions is the development and implementation of MCs that enhance biofertilization, stress tolerance, and crop yields. Nonetheless, the current level of adoption of innovative technologies remains low: fewer than 10% of agricultural producers utilize modern tools, including microbial products. This limited uptake stems from an underdeveloped extension and advisory infrastructure, a lack of robust technology transfer systems, and insufficient state support [108,109]. These issues are further compounded by chronically low funding for agricultural research, which lags significantly behind international benchmarks [110].

Despite these challenges, Kazakhstan has established a strong research foundation. Leading institutions include the Institute of Plant Biology and Biotechnology (IPBB), the National Center for Biotechnology (NCB), the Aitkhozhin Institute of Molecular Biology and Biochemistry, the Kazakh Research Institutes of Soil Science and Agrochemistry (KazNIIPiA), Plant Protection and Quarantine (KazNIIZiKR), and Microbiology and Virology. Major universities such as Nazarbayev University, Al-Farabi Kazakh National University, and L.N. Gumilyov Eurasian National University also actively contribute to agri-biotechnology R&D [111]. These institutions have developed and evaluated MCs with high efficacy under Kazakhstan’s agroecological conditions. One study assessed the effect of culture fluids from 36 collection strains and 16 rhizosphere isolates on wheat seed germination and growth, identifying the most active strains and forming three MCs. Consortium No. 1 (*Bacillus pumilis* Pol P3(1) 10 RKM0528, *Bacillus thuringiensis* Pb 30 RKM0341, *Bacillus licheniformis* 356 RKM0074, *Serratiamarcescens* Sh-2) and No. 2 (*Rhizobium legiminosarum* B-6 RKM 0272, *Azotobacterchrococcum* Azp24 B-RKM 820, *Bacillus pumilis* Pol P3(1) 10 RKM0528, *Serratiamarcescens* Sh-1) were most effective in promoting germination and seedling growth [112]. In field experiments in Northern Kazakhstan, the effects of four MCs on the grain yield of spring barley (‘Tselinny 2005’) were evaluated. The most effective were consortiums B1 (*Streptomyces sindenensis* PM9, *Streptomyces griseus* PM25, *Bacillus aryabhattai* PM62, *Bacillus aryabhattai* PM68, *Bacillus aryabhattai* PM69, *Bacillus megaterium* PM80B, *Lentzea violacea* PM86B) and B4 (*Trichoderma* strains T134, T115, and T200 were identified as representatives of *Tr. lignorum* and *Tr. album* species), which boosted yields by up to 50% compared to the control and exhibited tolerance to both biotic and abiotic stress [113]. A consortium composed of rhizobia and phosphate-solubilizing bacteria was developed to improve nitrogen and phosphorus nutrition in soybean (*Glycine max* (L.) Merr.) and increase yields. Consortium No. 21, which included *Rhizobium lupini* RH7 and *Pseudomonas koreensis* FT4, significantly increased root length, shoot height, nodule number, and plant protein content compared to the control [114]. Under saline stress on degraded soils, co-inoculation with salt-tolerant rhizobia and phosphate-solubilizing bacteria improved soybean (*Glycine max* (L.) Merr.) resilience. The combined inoculation of *Bradyrhizobium japonicum* RH28 and *Pseudomonas koreensis* FT4 resulted in a threefold increase in root biomass and nodule formation, as well as a 2.7-fold increase in shoot biomass compared with the control [115]. The ZOB-1 consortium, composed of *Anabaena variabilis, Chlorella vulgaris,* and *Azotobacter* sp., along with the ZOB-2 consortium, consisting of *Nostoc calcicola, Chlorella vulgaris*, and *Azotobacter* sp., were proposed for use in agrobiotechnology for rice cultivation with the aim of enhancing seed germination and enriching the soil with fixed nitrogen. Both consortia exhibited high photosynthetic activity and promoted seed germination and plant growth, with ZOB-1 being recommended as an effective biostimulant and biofertilizer for application in local agricultural practices [116].

However, the broader adoption of MCs in Kazakhstani agriculture is hindered by several regulatory, institutional, and market barriers. Despite their biological nature, microbial products with growth-promoting functions are regulated under strict pesticide laws, necessitating expensive and time-consuming registration processes, including field trials and toxicological assessments. This creates regulatory overlap, legal ambiguity, and a lack of formal classification for MCs. Legislative differentiation between biological products and chemical agents, as well as the creation of a dedicated registry for MCs with expedited certification, is urgently needed.

Another unresolved issue is the technology transfer gap. Research rarely reaches farmers due to the absence of institutional dissemination mechanisms, weak cross-sector coordination, a shortage of demonstration farms, underdeveloped advisory systems, and a lack of financial incentives for adoption. Additional challenges include low private-sector involvement, limited investor interest, and weak communication channels between science and production. International experience, including China’s model, shows that effective transfer systems rely on integrated networks of science, government, and cooperatives, where state programs provide subsidies, universities offer training, and demonstration farms showcase results.

Further barriers include fragmented regulatory documents, the absence of national standards for MCs, limited production capacity for scaling technologies, and a shortage of skilled specialists in microbial biotechnology and agroecology. Equally important is the insufficient level of international cooperation and weak representation of Kazakhstani developments on global biotechnology platforms.

Kazakhstan possesses all the prerequisites for large-scale adoption of MCs from scientific expertise to natural resource diversity. However, realizing this potential requires a systemic and cross-sectoral approach aimed at eliminating regulatory barriers, ensuring long-term funding, establishing knowledge transfer platforms, strengthening international scientific collaboration, and training qualified personnel. MCs must be viewed not as auxiliary tools but as a central component of a new agricultural policy, one that fosters an ecologically sustainable, technologically advanced, and food-secure model of 21st-century agriculture in Kazakhstan.

## 5. Technology Transfer in Agricultural Practice: The Cases of China and Kazakhstan

China’s domestic demand for plant-based commodities continues to expand, driven by population growth, urbanization, and the dietary shift toward higher-protein foods that require substantial feed grain and oilseed inputs. Although major investments in high-yield cultivars, irrigation modernization and “green” input subsidies have tempered the rate of import growth, the country is expected to remain a net buyer of wheat, maize, and soy-based products over the coming decade. By contrast, Kazakhstan’s comparatively small population and extensive arable land enable the country to produce a structural surplus of wheat and oilseed crops; these exports constitute a central pillar of national income but are vulnerable to drought-induced yield variability and logistical bottlenecks.

Both governments employ a similar policy mix to manage the balance between supply and demand. China relies on minimum-price procurement, strategic grain reserves, and differentiated tariffs to stabilize domestic markets, while Kazakhstan uses export duties and seasonal quotas to prevent local shortages and maintain price parity with international benchmarks. Each country also offers targeted subsidies for biological inputs and resource-efficient technologies that raise yields without escalating chemical use.

This alignment creates a complementary trade dynamic: China secures a geographically close source of high-quality wheat and oilseeds, whereas Kazakhstan gains preferential rail access to an immense consumer market and imports high-value seeds, fertilizers, and agricultural machinery from China. Looking ahead, sustained growth in Chinese feed demand and Kazakhstan’s commitment to expanding grain exports suggest a stable, mutually beneficial corridor for plant products—one that will be further strengthened by continued policy coordination on pricing, sanitary standards, and investment in cross-border logistics. For most developing countries, the transfer of agricultural technologies serves as a key mechanism for enhancing sustainability, ensuring food security, and facilitating the adoption of innovative solutions. Given the limited access to domestic R&D funding, emerging economies often rely on adapting foreign technologies, engaging in international knowledge exchange, implementing imitation strategies, and attracting foreign direct investment. However, the success of such approaches depends on the existence of an institutional environment capable of effectively integrating external technologies into domestic agri-production systems. In this context, China’s experience stands out: agricultural technology transfer is embedded within the country’s national strategy for scientific, technological, and food system development, supported in parallel by research institutions, development agencies, and the private sector [117].

**Figure 2:**
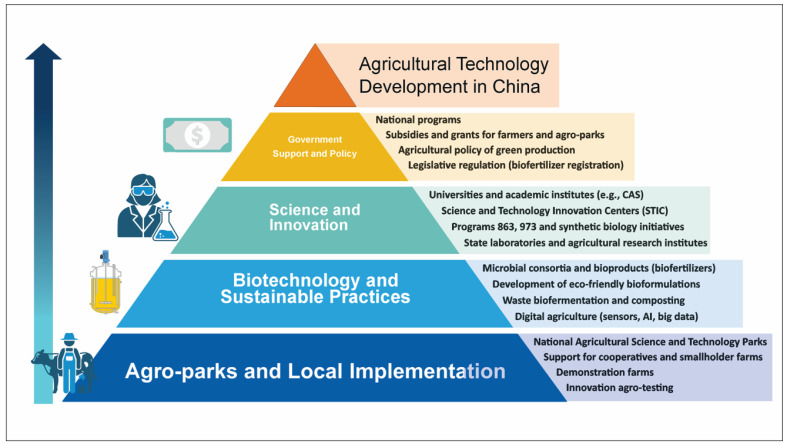
Structural framework of agricultural technology development factors in China.

China’s model of technology transfer is based on a consistent institutionalization of agro-innovation. As illustrated in Figure 2, the Chinese agro-innovation system follows a multi-tiered structure in which each level reinforces the next, forming a vertically integrated framework. At the top is the national strategy that sets the priorities for science and technology development in the agricultural sector. Implementation instruments include large-scale initiatives such as the Belt and Road Initiative and national programs to promote green agriculture, digitalization, and carbon footprint reduction. Government policy serves as a guiding force that defines the innovation trajectory and supports it through subsidies and tax incentives.

The next level is the institutional environment: agri-parks, industrial zones, and specialized biotech centers provide the infrastructure necessary for transforming scientific ideas into practical technologies. International cooperation platforms, such as COMSATS and CCIB, also play a pivotal role by fostering joint projects with universities and companies from dozens of countries. The third tier comprises the private sector, especially agribusinesses and technology startups, whose engagement in innovation is supported through public–private partnerships and driven by growing demand for high-value-added agricultural products. The sustainable interaction between business and science accelerates the commercialization and adaptation of technologies. The foundation of this system is its scientific and educational base—universities, research institutes, and laboratories, where knowledge, human capital, and applied solutions are generated. This strong foundation ensures not only autonomy in technological development but also China’s leadership in global agricultural technology transfer.

**Figure 3:**
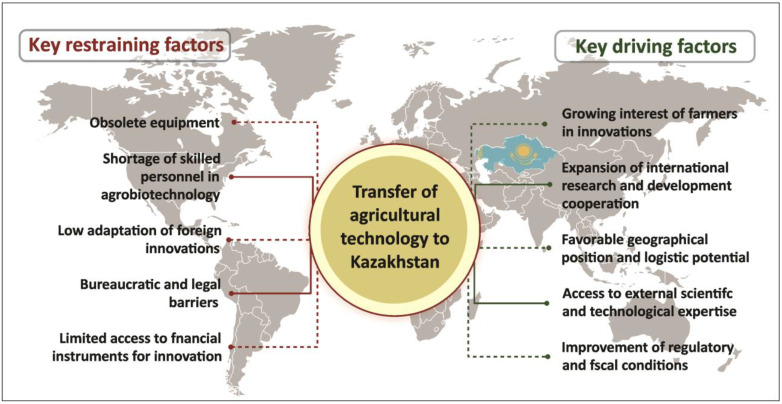
Key limiting and facilitating factors for the transfer of agrotechnologies in Kazakhstan.

In contrast, Kazakhstan is only beginning to build its own model of agricultural technology transfer. To visualize the key factors influencing this process, Figure 3 presents a force field diagram depicting the balance between facilitating and constraining elements. Green arrows represent driving forces, such as increasing farmer interest in innovation, expanding international collaboration, the country’s advantageous geographic position (with logistical benefits), support from international organizations for research, and improved access to external markets through reduced tax burdens. These drivers create favorable conditions for the adoption of technologies such as MCs and other biotechnological tools.

Conversely, red arrows illustrate restraining forces, including the physical and moral depreciation of agricultural equipment, outdated production technologies, a shortage of qualified personnel in agri-biotechnology, limited adaptability of foreign innovations to local contexts, and bureaucratic or legal hurdles. Additional constraints include inadequate sectoral funding and the high cost of leasing, which hinders the renewal of the technical base. The force field diagram underscores the structural tension in Kazakhstan between modernization efforts and persistent barriers that must be overcome for successful technology transfer.

Kazakhstan’s low level of technical infrastructure, limited human resources, and constrained financial capacity hinder the rapid development of agrotechnologies. While China benefits from dynamic cooperation between universities, research institutions, and businesses, Kazakhstan still lacks well-developed mechanisms for adapting foreign technologies and commercializing domestic innovations.

Nonetheless, bilateral cooperation between China and Kazakhstan rooted in historical ties and mutual benefit has gained new momentum under the Belt and Road Initiative. Cross-border flows of technology, capital, and knowledge have accelerated through bilateral agreements and joint projects, particularly in agribusiness [118]. One notable example is the participation of Kazakh researchers in the biotechnology initiatives of the Tianjin Institute of Industrial Biotechnology. Research on synthetic microbial fertilizers and biopesticides under the Tianjin SynBio program illustrates how scientific knowledge is being transferred into practice at the intersection of both countries’ strategic interests [119]. These projects contribute to increased ecological sustainability but also strengthen institutional collaboration in research and development.

Technology transfer is a complex process influenced by multiple factors ranging from political will to workforce competency. In Kazakhstan, the primary obstacles are economic and institutional in nature: bureaucracy, an insufficient legal framework, limited state support, and outdated agricultural infrastructure. Key growth points include improving workforce skills, expanding R&D funding, developing mechanisms for local adaptation of technologies, and establishing infrastructure for innovation commercialization [120]. Currently, there is a steady increase in demand for plant products, especially cereals such as wheat, in China, which creates favorable conditions for increasing exports from Kazakhstan. To regulate the interstate trade balance, both countries apply export and import quotas, mutual supply agreements, and investments in joint agricultural projects. Kazakhstan has already launched initiatives to expand agricultural exports to China, but this process is hindered by technical regulation differences, logistical limitations, and institutional fragmentation [121].

To ensure the successful transfer of agricultural technologies, a comprehensive improvement in conditions is required from the harmonization of standards and the establishment of a knowledge exchange system to the development of human resource and information support services. Coordinated efforts between government, business, and academia should be complemented by a multi-level infrastructure that covers the entire innovation lifecycle, from laboratory research to large-scale field implementation. The Chinese model may serve as a reference point for building a national agricultural technology transfer system in Kazakhstan, adapted to local socioeconomic and institutional contexts.

Against this backdrop, the microbial fertilizer market illustrates how policy incentives can be translated into tangible commercial demand. Even with partial application across Kazakh farmland, the demand for such products is expected to be substantial. A phased strategy appears most appropriate: initially, the import of proven MCs from China; next, the establishment of joint blending and packaging facilities; and finally, the development of full-scale domestic production as technical expertise and market stability grow. This model ensures rapid product availability, reduces dependency on a single supplier, and simultaneously enhances farm-level economic efficiency while improving soil health.

The successful outcomes of laboratory and small-scale field trials of microbial fertilizers are not always reproducible at a larger scale, highlighting the need for a comprehensive assessment of their economic feasibility, adaptation to specific agroecological conditions, and the readiness of farmers to adopt new agricultural technologies. Farmers remain a critical link in this process, as their perception of innovations and practical engagement largely determine the success of the transition to sustainable farming practices. The higher initial costs of microbial inputs compared to conventional chemical fertilizers may lead to skepticism or resistance among farmers. In this context, strategic state involvement is essential through financial incentives, the development of agricultural education and extension systems, as well as the promotion of international cooperation and technology transfer, as exemplified by the experiences of China and Kazakhstan. Strengthening bilateral scientific collaboration, expanding knowledge exchange, and enhancing cross-sectoral coordination will significantly accelerate the adoption of innovative agrotechnologies. Ultimately, these measures will not only improve food security in both countries but also enhance their roles in global agricultural production and supply chains.

## 6. Conclusions

MCs, as the foundation of biofertilizers, represent a key component in transforming modern agriculture toward more sustainable, environmentally friendly, and productive systems. The comparative analysis of China and Kazakhstan has revealed both shared and unique aspects of their approaches to developing, deploying, and scaling biotechnological solutions. China’s experience characterized by a strong research infrastructure, consistent governmental support, and a well-established technology transfer system demonstrates a mature model for integrating MCs into agricultural practice. In contrast, Kazakhstan is still in the process of forming its biotechnological policy. However, the country holds considerable potential, with a solid scientific base, agroecological diversity, and increasing farmer interest in organic technologies. Effective adaptation of China’s experience requires consideration of Kazakhstan’s specific institutional, regulatory, and climatic conditions, as well as overcoming existing barriers. A strategic priority should be the development of a national agro-innovation system built on inter-agency collaboration, the establishment of demonstration farms, a strengthened role for universities, and support for R&D. MCs should be seen not only as a technological tool but also as an institutional bridge between countries with different development levels, enabling mutual exchange of knowledge, technologies, and resources. Establishing a multi-level innovation ecosystem adapted to local conditions is essential for improving agricultural productivity, restoring degraded land, and enhancing food security in an era of global change.

## Figures and Tables

**Table 1:** Microbial consortiums enhancing plant resistance to biotic stress.

№	Consortium Composition	Host Crop	Key Effect	Reference
1	*Asticcacaulis* sp. + *Arachidicoccus* sp. + *Phenylobacterium* sp.	Tomato	Reliable protection against *Botrytis cinerea* via induced immunity and biofilm formation	[69]
2	*Bacillus cereus* AR156 + *B. subtilis* SM21 + *Serratia* sp. XY21	Cucumber	Nematode-induced root galls reduced by 56–72%; improved yield and rhizosphere health	[70]
3	*Rhodotorula graminis* JJ10.1 + *Pseudomonas psychrotolerans* YY7 + *P. chlororaphis* T8 + *Bacillus amyloliquefaciens* FZB42	*Arabidopsis thaliana* and tomato	Cross-kingdom consortium prevented bacterial–fungal infections and promoted growth via synergistic biofilm	[71]
4	*Bacillus cereus* BT-23 + *Lysobacter antibioticus* 13-6 + *L. capsici* ZST1-2	Chinese cabbage	Kiel’s disease caused by *Plasmodiophora brassicae* is reduced reduced by ~66%; increased yield and reduced soil acidity	[72]
5	*Trichoderma asperellum* GDSF1009 + *T. asperelloides* Z4-1 + *T. harzianum* 10569 + *T. asperellum* 10264	Cucumber	Suppressed *Fusarium* wilt; enhanced seedling growth and amino acid accumulation compared to monocultures	[73]
6	*Lysobacter enzymogenes* OH11W (ΔWAP-8294A2) + *Bacillus safensis* ZK-1	Kiwi	Controlled bacterial canker and associated fungal infections	[74]
7	*Bacillus subtilis* 503 + *B. safensis* 537 + *B. amyloliquefaciens* 337 + *B. sonorensis* 544	Ginkgo	Field control of leaf blight up to 100%; increased biomass and antioxidant activity, and improved soil microbiota	[75]

**Table 2:** Microbial consortiums enhancing plant tolerance to abiotic stress.

**№**	**Consortium Composition**	**Host Crop**	**Key Effect**	**Reference**
*Heavy metals*				
1	*Bacillus cereus* + *B. thuringiensis* + *Herbaspirillum huttiense*	Wheat	Reduced available Pb/Cd in soil and shoot translocation; enhanced root development	[81]
2	*Cellulomonas iranensis* ZJW-6 + *Pseudomonas brassicacearum* wj1	Rice	Removed 94% Cd and 74% Pb in 7 days; improved soil structure and adsorption	[82]
3	*Bacillus subtilis* SQ4 + *Enterobacter hormaechei* VY5 + *B. velezensis* SQ6	Sorghum	Alleviated combined PVC + Cd stress; increased dry biomass and nutrient availability	[83]
4	*Paenibacillusmucilaginosus* ACCC10013 +* Sinorhizobium meliloti* CCNWSX0020	Alfalfa	Mitigated Cu toxicity by reducing ROS and lipid peroxidation	[84]
5	*Enterobacter bugandensis* XY1 + *Serratia marcescens* X43	Water spinach	Reduced Cd and Pb in aboveground biomass by 51–80% via polyamine-mediated immobilization	[85]
6	*Leptolyngbya* sp. XZMQ + *Bacillus* XZM	Sunflower	Reduced As in roots/stems/leaves by 38–70%; enhanced soil enzymatic activity	[86]
*Salinity*				
7	*Pseudomonas* sp. P8 + *Peribacillus* sp. P10 + *Streptomyces* sp. X52	Maize	Improved growth and enriched nitrogen fixers in rhizosphere under salinity stress	[87]
8	*Azotobacter beijerinckii B3* + *Chlorella pyrenoidosa*	Wheat	Increased biomass by 67% under alkaline stress; reduced pH, enhanced fertility	[88]
9	*Paenibacillus sabinae* + *Leptolyngbya* sp. RBD05	Wheat	Co-inoculation increased dry weight by 85% and K:Na ratio by 41%; improved salt tolerance	[89]
10	*Bacillus licheniformis* (NX-3/59) + *B. subtilis* (NX-4/48/62)	Cucumber (seedling)	Enhanced stem diameter and fresh weight under salt stress by activating substrate nutrients	[90]
11	11-strain-SMC from *Kalidium schrenkianum*	Wheat	Stimulated germination, antioxidant enzymes, and chlorophyll; reduced oxidative stress	[91]
*Drought*				
12	*Burkholderia* sp. UWIGT-83 + *Burkholderia* sp. UWIGT-120	Red hot pepper	Improved germination and growth under simulated drought via ACC deaminase and biofilm	[92]
13	*Bacillus cereus* JQB1 + *Rhodococcus sphaeroides* JQB3 + *Serendipita indica* JQF1 + *Mortierella alpina* JQF2 + *Ceriporia lacerata* JQF3 + *Fusarium equiseti* JQF4	Tartary buckwheat	Increased biomass, photosynthesis, and reduced H_2_O_2_/MDA; improved drought tolerance	[93]

## Data Availability

Not applicable.

## Abbreviations

The following abbreviations are used in this manuscript:

MC	Microbial Consortium
R&D	Research and Development
PGPMs	Plant Growth-Promoting Microorganisms
HMs	Heavy Metals
